# Cost-Effective Emission Abatement in Europe Considering Interrelations in Agriculture

**DOI:** 10.1100/tsw.2001.295

**Published:** 2001-10-30

**Authors:** Corjan Brink, Ekko van Ierland, Leen Hordijk, Carolien Kroeze

**Affiliations:** National Institute of Health and the Environment (RIVM), Bilthoven, The Netherlands

**Keywords:** environmental policy interrelations, emission abatement, cost-effectiveness, acidification, greenhouse gases

## Abstract

Agriculture is an important source of ammonia (NH3), which contributes to acidification and eutrophication, as well as emissions of the greenhouse gases nitrous oxide (N2O) and methane (CH4). Controlling emissions of one of these pollutants through application of technical measures might have an impact (either beneficial or adverse) on emissions of the others. These side effects are usually ignored in policy making. This study analyses cost-effectiveness of measures to reduce acidification and eutrophication as well as agricultural emissions of N2O and CH4 in Europe, taking into account interrelations between abatement of NH3, N2O, and CH4 in agriculture. The model used is based on the RAINS (Regional Air pollution INformation and Simulation) model for air pollution in Europe, which includes emissions, abatement options, and atmospheric source-receptor relationships for pollutants contributing to acidification and eutrophication. We used an optimisation model that is largely based on the RAINS model but that also includes emissions of N2O and CH4 from agriculture and technical measures to reduce these emissions. For abatement options for agricultural emissions we estimated side effects on other emissions. The model determines abatement strategies to meet restrictions on emission and/or deposition levels at the least cost. Cost-effective strategies to reduce acidification and eutrophication in Europe were analysed. We found that NH3 abatement may cause an increase in N2O emissions. If total agricultural N2O and CH4 emissions in Europe were not allowed to increase, cost-effective allocation of emission reductions over countries in Europe changed considerably.

### INTRODUCTION

Many European countries agreed to reduce emissions contributing to acidification, eutrophication, and ground-level ozone (Gothenburg Protocol[1]) and greenhouse gas emissions (Kyoto Protocol[2]). Interrelations exist between policies for reducing emissions of air pollutants and greenhouse gases[3]. Several recent studies focus on ancillary benefits of efforts to reduce $CO_2$ emissions from fossil fuel use[4]. In earlier work, we showed the existence of interrelations between acidification and greenhouse gas mitigation policies in European agriculture[5,6,7].

For greenhouse gases, the location of emissions is not important for their environmental impact. For pollutants contributing to acidification and eutrophication, however, the environmental impact depends on emission location and atmospheric transport before deposition. Moreover, the same level of deposition may have different environmental impacts at different locations because of differences in ecosystem sensitivities. A consideration of the side effects of air pollutants on emissions may therefore change the cost-effective geographical distribution of greenhouse gas emission reductions[8].

In this study we extend earlier work on interrelations between abatement of ammonia ($NH_3$), nitrous oxide ($N_2O$), and methane ($CH_4$) from European agriculture[5,6,7]. Here we also consider emissions of acidifying compounds other than $NH_3$ (namely sulphur dioxide [$SO_2$] and nitrogen oxides [$NO_x$]), the location of emissions and environmental effects and atmospheric transport of emissions in between, and environmental sensitivity of various locations. We analyse the impact of interrelations among abatement policies in agriculture on the cost-effective allocation of abatement over sources of emissions.

### MODEL

A comparative static optimisation model was used to determine cost-effective abatement strategies in Europe for acidification, eutrophication, and agricultural emissions of $N_2O$ and $CH_4$ simultaneously, considering interrelations between abatement activities in the agricultural sector. The model is largely based on the RAINS (Regional Air pollution INformation and Simulation) model developed at the International Institute for Applied Systems Analysis as a tool for integrated assessment of alternative strategies to reduce air pollution in Europe[9,10]. The RAINS model includes data to estimate emissions of $SO_2$, $NO_x$, and $NH_3$ in Europe, as well as a large number of technical measures to abate these emissions. The model we used for this analysis is described in detail in Brink et al.[11]. This section will briefly describe this basic model and present some extensions to include several environmental effects and atmospheric transport of pollutants from the location of emissions to the location of the environmental impact.

#### Basic Model

The model is used to calculate emissions of various pollutants and determine cost-effective abatement strategies for restrictions on emission levels. Unabated emissions are calculated from (exogenous) data on various economic activities and can be reduced by applying abatement options at various sources. The model minimises total cost of abatement:

$$\min \sum_{k \in K} C_k\left(v_k\right) \qquad (1)$$

where $v_k$ is a vector with elements $a_{k,n}$ ($n \in N$);

$$C_k\left(v_k\right) = \sum_{n \in N} a_{k,n} \cdot \bar{X}_k \quad \forall\, k \in K \qquad (2)$$

subject to

$$\sum_{k \in K} e_{k,p} \le \bar{E}_p \quad \forall\, p \in P, \qquad (3)$$

$$e_{k,p} = \varepsilon_{k,p} \cdot \bar{X}_k \cdot \left(1 - \sum_{n \in N} a_{k,n} \cdot \rho_{k,n,p}\right) \forall\, p \in P, k \in K, \qquad (4)$$

$$\sum_{n \in N} a_{k,n} \le 1 \quad \forall\, k \in K, \quad \text{and} \qquad (5)$$

$$a_{k,n} \ge 0 \quad \forall\, k \in K, \quad n \in N. \qquad (6)$$

Decision variables in the model are application rates ($a_{k,n}$) for abatement options ($n$) at source $k$ (lower case letters indicate individual elements of sets and upper case letters indicate sets). Abatement costs at source $k$ ($C_k$) depend on the (exogenous) activity level ($\bar{X}_k$) and on application rates ($a_{k,n}$) and per unit costs ($\gamma_{k,n}$) for all abatement options ($n \in N$) (Eq. 2). Annual emissions of pollutant $p$ from source $k$ ($e_{k,p}$) depend on activity level ($\bar{X}_k$), emission factor ($\varepsilon_{k,p}$), application rate ($a_{k,n}$), and effectiveness ($\rho_{k,n,p}$) of abatement options (Eq. 4). An upper level for emissions of each pollutant $p$ is indicated by $\bar{E}_p$ (Eq. 3) and application rates ($a_{k,n}$) are constrained in Eq. 5 and 6.

Emissions can be reduced by applying add-on abatement techniques. For each source, the model chooses application rates for all abatement options included in set $N$ such that emission targets are obtained at minimum cost. Abatement options are not attributed to a specific pollutant; instead, an option may have impacts on various pollutants, either reducing or increasing emissions, but reducing emissions of at least one pollutant. The effect of abatement option $n$ on emissions of pollutant $p$ from source $k$ is given as the fraction of unabated emissions that is reduced ($\rho_{k,n,p}$). Emissions of pollutant $p$ can be reduced ($0 < \rho_{k,n,p} \le 1$; i.e., a reduction to a maximum of 100%), unaffected ($\rho_{k,n,p} = 0$), or increased ($\rho_{k,n,p} < 0$; i.e., an increase can be more than 100%). The changes in emissions by abatement option $n$ at source $k$ depend linearly on the level of unabated emissions and on the application rate ($\varepsilon_{k,p} \times \bar{X} \times a_{k,n} \times \rho_{k,n,p}$).

There may be interaction between different measures to reduce emissions. For instance, measures may exclude, reinforce, or weaken each other, or some measures can only be applied in combination with others. To take this into consideration, possible combinations of measures and their combined effect were included as new abatement options. This may result in a large number of abatement options included in set $N$. Moreover, a certain level of detail about sources and abatement options is required in the model because side effects of emission-reduction measures may depend on specific characteristics of sources and measures. As a result, the model includes a large number of variables. To be able to solve such a large problem, the model applies a (stepwise) linear approach to calculate emissions, costs, and effects of abatement.

In the optimisation procedure, the abatement options that are applied for specific emission constraints are determined. Costeffectiveness of abatement strategies depends on the cost of abatement options, the magnitude of effects of abatement options on emissions of various pollutants, and reduction targets for emissions of various pollutants.

### Multiple Environmental Targets

To reflect the contribution of various pollutants to different types of environmental damage, the model includes indicators for environmental problems ($q \in Q$), reflecting the size of the environmental impact. These indicators depend linearly on the emissions of pollutants.* The indicator for environmental problem $q$ ($h_q$) is determined by:

$$h_q = \sum_{k \in K} \sum_{p \in P} \psi_{p,q} \cdot e_{k,p} \qquad (7)$$

where $\psi_{p,q}$ represents the contribution of one unit of pollutant $p$ to environmental problem $q$ in terms of the indicator used for this environmental problem (for example, $\psi_{p,q}$ may be the global warming potential [GWP] for greenhouse gas $p$, indicating the relative contribution of this pollutant to global warming). Constraints in the model can be specified in terms of these indicators:

$$h_q \le \bar{H}_q \quad \forall\, q \in Q \qquad (8)$$

where $\bar{H}_q$ indicates the target level for indicator $h_q$.

### Location of Emissions and Effects

Emissions occur at different locations. For some pollutants (e.g., air pollutants) the environmental impact depends on the location of the emissions and transport of pollutants through the atmosphere, whereas for others (e.g., greenhouse gases) the location does not matter because emissions are uniformly mixing in the atmosphere. In the case of air pollutants, the environmental impact does not necessarily occur at the place of emissions because pollutants may be transported through the atmosphere over long distances before they have an effect on environmental quality[9]. For pollutants of this type, it is important to take into account the location of emissions, the atmospheric transport, and the location of the environmental effect when determining cost-effective abatement strategies. To this end, the model is extended to include a set $I$ of source locations (i.e., where the emissions occur) and a set $J$ of receptor locations (i.e., where the environmental effects occur).** All sources (and hence emissions) are assigned to a source location ($i \in I$). Consequently, the dimension $i$ is added to economic activity level ($\bar{X}_k$), emission level ($e_{k,p}$), and application rate ($a_{k,n}$) (i.e., they become $\bar{X}_{i,k}$, $e_{i,k,p}$, and $a_{i,k,n}$, respectively). Because of different characteristics of sources at different locations, both the emission factors and the parameters for the cost and effect of abatement options are also location specific (i.e., $\varepsilon_{k,p}$, $\gamma_{k,n}$, and $\rho_{k,n,p}$ are replaced by $\varepsilon_{i,k,p}$, $\gamma_{i,k,n}$, and $\rho_{i,k,n,p}$, respectively). In accordance with these changes, Eq. 1, 2, 4, 5, and 6 are respectively replaced by

$$\min \sum_{i \in I} \sum_{k \in K} C_{i,k}\left(v_{i,k}\right) \qquad (9)$$

where $v_{i,k}$ is a vector with elements $a_{i,k,n}$ ($n \in N$);

$$C_{i,k}\left(v_{i,k}\right) = \sum_{n \in N} a_{i,k,n} \cdot \gamma_{i,k,n} \cdot \bar{X}_{i,k}; \qquad (10)$$

$$e_{i,k}, p = \varepsilon_{i,k,p} \cdot \bar{X}_{i,k} \cdot \left(1 - \sum_{n \in N} a_{i,k,n} \cdot \rho_{i,k,n,p}\right); \qquad (11)$$

$$\sum_{n \in N} a_{i,k,n} \le 1 \quad \forall\, i \in I, k \in K; \text{ and} \qquad (12)$$

$$a_{i,k,n} \ge 0 \quad \forall\, i \in I,\ k \in K,\ n \in N. \qquad (13)$$

The process of atmospheric transport of pollutants is included in the model, assuming a linear relationship between emissions of pollutant $p$ at source location $i$ that are deposited at receptor location $j$. The constant parameter $\tau_{i,j,p}$ represents the fraction of emissions of pollutant $p$ at $i$ that is deposited at $j$. The same approach is used in the RAINS model to include atmospheric transport of air pollutants[9,10]. Restrictions can be specified for emissions and environmental effects at various (source or receptor) locations or for overall emissions and environmental effects.

## COST-EFFECTIVE ABATEMENT IN EUROPE

The model was used to analyse cost-effective policies to reduce acidification and eutrophication in Europe, as well as emissions of greenhouse gases such as $N_2O$ and $CH_4$ from agriculture, considering interrelations between technical abatement measures in the agricultural sector. Acidification in Europe is mainly the result of emissions of $SO_2$, $NO_x$, and $NH_3$. Both $NO_x$ and $NH_3$ also play an important role in eutrophication. $SO_2$ and $NO_x$ emissions are to a large extent caused by fossil fuel combustion, whereas the major source of $NH_3$ is agriculture. Important gases contributing to global warming are carbon dioxide ($CO_2$), $N_2O$, and $CH_4$. In this study only greenhouse gas emissions related to agricultural activities are included. In Europe, $N_2O$ and $CH_4$ are to a large extent the result of agricultural activities. In the 1990s, agriculture was responsible for about 8% of total greenhouse gas emissions in Europe[12]. European agriculture is a minor source of $CO_2$ emissions, but may play a role in reducing $CO_2$ emissions by replacing fossil fuels with biomass and by removing $CO_2$ through sinks[13]; however, these considerations are not included in this analysis.

* This is an accepted approach for some environmental problems, such as acidification, and for adding up emissions of various greenhouse gases. For other environmental problems, such as the formation of ground-level ozone, which is the result of a nonlinear relationship between several pollutants, this approach is not possible. In principle, this kind of nonlinear relationship can be included in the model using a stepwise linear approach.

** Both source [$i$] and receptor [$j$] locations can be countries, but a higher or lower level of aggregation is possible as well. Moreover, the level of aggregation is not necessarily the same for source and receptor.

### Application of the Model

Environmental problems included in the model (set $Q$) are acidification, eutrophication, and greenhouse gas emissions; only emissions of $N_2O$ and $CH_4$ from agriculture are considered. Pollutants considered (set $P$) are $SO_2$, $NO_x$, $NH_3$, $N_2O$, and $CH_4$. For acidification and eutrophication, the analysis considers atmospheric transport of pollutants before they are deposited. Moreover, targets for acidification and eutrophication take into account ecosystem sensitivity at the location of deposition. For $N_2O$ and $CH_4$ from agriculture, restrictions are on the sum of $CO_2$-equivalent emissions of these gases,* which is justified because $N_2O$ and $CH_4$ are uniformly mixed in the atmosphere. Indicators for the environmental impacts ($h_q$) are acid equivalents (acidification), nitrogen (eutrophication), and $CO_2$-equivalents (greenhouse gas emissions).

Furthermore, the model includes emissions from 36 European countries (set $I$). For $SO_2$, $NO_x$, and $NH_3$, the model considers atmospheric transport of emissions from each country to about 500 grids of 150 × 150 km in Europe (set $J$). The source-receptor relationship ($\tau_{i,j,p}$, indicating the fraction of pollutant $p$ emitted in country $i$ that is deposited in grid $j$) was taken from the RAINS model. Source-receptor matrices in the RAINS model were derived from a model of long-range transport of air pollution developed by EMEP (the Cooperative Programme for Monitoring and Evaluation of the Long-Range Transmission of Air Pollutants in Europe)[10,14].

Within each country, the model distinguishes various sources of emissions (set $K$). The focus of this study is on the agricultural sector; therefore, agricultural activities (as sources of $NH_3$, $N_2O$, and $CH_4$) are considered in more detail than nonagricultural activities (as sources of $SO_2$ and $NO_x$). For agriculture, the model includes 14 sources of emissions in each country (viz. 11 animal categories, 2 types of fertiliser used, and production of nitrogen fertilisers). For $SO_2$ and $NO_x$, all sources in a country were aggregated in one source for $SO_2$ and one source for $NO_x$ emissions (with activity levels $\bar{X}_{i,k}$ for these sources representing emissions of $SO_2$ and $NO_x$). Activity levels for the various sources in each country ($\bar{X}_{i,k}$) and the associated emission factors for $NH_3$ ($\varepsilon_{i,k,NH3}$) were taken from databases in the RAINS model. This model includes data for 1990, 1995, and projections up to 2010. Agricultural emissions of $N_2O$ and $CH_4$ were estimated on the basis of the databases in the RAINS model using emission factors as described in Brink et al.[5].

The model includes a set $N$ of abatement options. Information on costs per unit of activity ($\gamma_{i,k,n}$) and effects on emissions ($\rho_{i,k,n,p}$) was taken from the RAINS model[10,15] and from studies for the EU[16,17]. For abatement options applicable to agricultural activities, we estimated effects on other emissions[5,7].

For each grid cell, we used the fifth percentiles of the critical loads of sulphur and nitrogen (both acidifying and nutrient).** These values were calculated using critical loads for many ecosystems that occur within a grid cell[19,20]. If the deposition in a grid cell does not exceed these values, 95% of the ecosystem area within the grid cell is protected.

Emissions and abatement costs in 2010 were estimated for four scenarios. First, the no-control (NOC) scenario assumes no technical abatement measures applied in any European country (this scenario also excludes measures that have to be taken in the future according to adopted national and international legislation for emission control). Second, the 40% gap-closure (GAP) scenario reflects a reduction in emissions of acidifying compounds in Europe such that for each grid cell, the critical load exceedance*** for acidification and eutrophication in the NOC scenario is reduced by at least 40% at minimum total abatement cost. Third, the 40% gap-closure-plus ($GAP^+$) scenario requires a 5% reduction in the sum of $N_2O$ and $CH_4$ emissions (in $CO_2$-equivalents) from agriculture in Europe relative to their NOC levels by measures additional to the abatement options applied in the cost-effective solution in the GAP scenario. Finally, the 40% gap-closure double-plus ($GAP^{++}$) scenario combines the 40% gap-closure requirement for acidification and eutrophication with the 5% reduction requirement for $N_2O$ and $CH_4$ emissions (in $CO_2$-equivalents) from agriculture in Europe. In the $GAP^{++}$ scenario, cost-effective abatement strategies are determined for both the 40% gap-closure and the 5% emission-reduction requirements simultaneously, considering interrelations and side effects of abatement options in agriculture.

### RESULTS AND DISCUSSION

To achieve the reduction in critical-load exceedance, emissions of $SO_2$, $NO_x$, and $NH_3$ have to be reduced considerably (Table1). $NH_3$ abatement without restrictions on $N_2O$ and $CH_4$ emissions (GAP) caused an increase in total $N_2O$ emissions from European agriculture of 8% relative to NOC (Table 1) as a result of negative side effects of $NH_3$ abatement options. Total agricultural emissions of $CH_4$ decrease a little as a result of the side effects of $NH_3$ abatement. The net increase in $N_2O$ and $CH_4$ emissions from agriculture in $CO_2$-equivalents is 4%.

Adding a reduction target for $N_2O$ and $CH_4$ emissions from European agriculture ($GAP^+$ and $GAP^{++}$) obviously results in higher total abatement costs than in the GAP scenario (Table 1). Results for $GAP^+$ are different from $GAP^{++}$ because $GAP^+$ analyses optimal abatement strategies for acidification and greenhouse gases separately; $GAP^{++}$ reflects an integrated approach reducing acidifying emissions and agricultural $N_2O$ and $CH_4$ emissions simultaneously, considering interrelations between abatement of $NH_3$, $N_2O$, and $CH_4$ in European agriculture. In $GAP^+$, the GAP abatement strategy is maintained, including abatement options that cause an increase in $N_2O$ emissions. The required reduction in $N_2O$ and $CH_4$ can only be obtained by applying additional abatement options so that the reduction target for $N_2O$ and $CH_4$ is obtained without violating restrictions for acidification and

* $N_2O$ and $CH_4$ emissions are converted to $CO_2$-equivalent emissions using the Intergovernmental Panel on Climate Change's GWPs for a time horizon of 100 years [310 for $N_2O$; 21 for $CH_4$].

** A critical load for acidification and eutrophication has been defined as "a quantitative estimate of an exposure to one or more pollutants below which significant harmful effects on specified sensitive elements of the environment do not occur according to present knowledge.„[18,19].

*** A critical load exceedance is defined as "the sum of the nitrogen and sulphur deposition reduction required to reach the critical load function by the 'shortest' path.„[19].

**TABLE 1**
**Estimated Emissions and Total Abatement Cost in Europe in 2010 for Four Scenarios (NOC, GAP, GAP$^+$, and GAP$^{++}$)**

| | NOC | GAP | GAP$^+$ | GAP$^{++}$ |
|---|---|---|---|---|
| $SO_2$ | 15.9 (100%) | 12.4 (78%) | 12.4 (78%) | 12.4 (78%) |
| $NO_x$ | 16.7 (100%) | 13.6 (82%) | 13.4 (80%) | 12.9 (78%) |
| $NH_3$ | 6.7 (100%) | 4.8 (72%) | 4.9 (73%) | 4.9 (74%) |
| $N_2O$[a] | 1.7 (100%) | 1.8 (108%) | 1.6 (95%) | 1.6 (95%) |
| $CH_4$[a] | 16.6 (100%) | 16.3 (98%) | 15.7 (95%) | 15.6 (94%) |
| Abatement cost[b] | | 41.7 | 55.8 | 47.6 |

*Note:* Emissions are in million tons $SO_2$, $NO_2$, $NH_3$, $N_2O$, and $CH_4$ /year; abatement costs are in billion $Euros_{1990}$/year; percentages given represent emissions relative to emissions in NOC scenario.

[a] Emissions from agricultural activities only.

[b] Total cost for technical measures to reduce emissions in Europe.

eutrophication. In GAP$^{++}$, total abatement costs are 15% lower than in GAP$^+$ because in GAP$^{++}$, it is possible to apply abatement options that simultaneously reduce $NH_3$ and $N_2O$ emissions instead of abatement options that reduce $NH_3$ emissions but cause a simultaneous increase in $N_2O$ emissions. The cost-effective allocation of abatement over countries also differs between GAP$^+$ and GAP$^{++}$ (Table 2). In particular, the change in abatement costs in GAP$^{++}$ relative to GAP$^+$ differs between countries. For most countries we calculated lower total abatement costs in GAP$^{++}$ than in GAP$^+$, but in some countries (e.g., Belgium and Finland) calculated costs are higher in GAP$^{++}$ than in GAP$^+$ (Table 2).

### CONCLUSIONS

Interrelations between policies aiming at different environmental problems may have an effect on cost-effective strategies to meet reduction targets for these environmental problems. This paper describes a stepwise linear, comparative, static optimisation model that can be used to analyse cost-effective emission reduction strategies to meet various environmental targets, considering several pollutants, atmospheric transport of pollutants, a large number of abatement options, and their effects on emissions of several pollutants.

The model was used to analyse cost-effective strategies to reduce acidification and eutrophication in Europe on the basis of information from the RAINS model. Moreover, we analysed the impact of $NH_3$ abatement on emissions of greenhouse gases such as $N_2O$ and $CH_4$ from agricultural activities. For abatement options applicable to agricultural activities, we estimated possible side effects on emissions. Reduction targets were specified for nitrogen and sulphur deposition exceeding critical loads for acidification and eutrophication in grid cells of 150 ↔ 150 km over Europe. We calculated a cost-effective allocation of abatement over sources to meet these targets with and without a restriction on emissions of $N_2O$ and $CH_4$. Without a restriction on $N_2O$ and $CH_4$ emissions, $N_2O$ emissions were calculated to increase 8% as a result of $NH_3$ abatement. A 5% reduction target for agricultural $N_2O$ and $CH_4$ emissions in Europe in addition to the acidification and eutrophication targets resulted in higher total abatement costs. We analysed a separate approach and an integrated approach to achieve the reduction targets for acidification and eutrophication and the reduction target for $N_2O$ and $CH_4$ emissions. The separate approach first determines a cost-effective abatement strategy for acidification and eutrophication targets only; this approach is maintained when subsequently an additional abatement strategy is determined to achieve the required reduction in $N_2O$ and $CH_4$ emissions. The integrated approach determines a cost-effective abatement strategy for all targets simultaneously. $NH_3$ abatement options that involve an increase in $N_2O$ emissions are maintained in the separate approach. In the integrated approach, however, it is possible to apply $NH_3$ abatement options without an effect on $N_2O$ or abatement options that simultaneously reduce $N_2O$ and $NH_3$ emissions instead of $NH_3$ abatement options causing an increase in $N_2O$. Consequently, total abatement costs are 15% lower in the integrated approach than in the separate approach. Moreover, the allocation of abatement costs over countries is different, with most countries facing lower costs with the integrated approach than with the separate approach, but with some countries facing higher costs.

This study focuses on interrelations between acidification and greenhouse gas mitigation policies in the agricultural sector, considering technical emission-control options. Interrelations between these policies may, however, also exist in other sectors, and other areas of policy-making may be interrelated with these policies. Moreover, in addition to the technical control options, structural changes may play an important role in reducing green-

**TABLE 2**
**Differences in Country-Specific Emissions and Abatement Costs between GAP$^{++}$ and GAP$^{+}$ Scenarios[a,b]**

| | $NO_x$ (GAP$^{++}$–GAP$^{+}$) | | $NH_3$ (GAP$^{++}$–GAP$^{+}$) | | $N_2O$[c] (GAP$^{++}$–GAP$^{+}$) | | $CH_4$[c] (GAP$^{++}$–GAP$^{+}$) | | Abatement Cost[d] (GAP$^{++}$–GAP$^{+}$) | |
|---|---|---|---|---|---|---|---|---|---|---|
| Albania | 0 | (1%) | 0 | (0%) | –0 | (0%) | –1 | (–1%) | 0 | (2%) |
| Austria | — | | 0 | (0%) | –1 | (–5%) | 4 | (2%) | –19 | (–4%) |
| Belarus | –10 | (–6%) | –3 | (–3%) | 2 | (6%) | –3 | (–1%) | –290 | (–30%) |
| Belgium | –8 | (–6%) | –2 | (–3%) | –1 | (–5%) | –5 | (–2%) | 186 | (8%) |
| Bosnia Herzegovina | –8 | (–16%) | 0 | (0%) | –0 | (–1%) | 0 | (0%) | 9 | (12%) |
| Bulgaria | –1 | (0%) | 1 | (1%) | 1 | (4%) | –2 | (–2%) | –296 | (–75%) |
| Croatia | –9 | (–17%) | 1 | (4%) | 0 | (2%) | 0 | (0%) | –65 | (–27%) |
| Czech Republic | — | | 1 | (1%) | 1 | (5%) | –6 | (–2%) | –278 | (–15%) |
| Denmark | — | | –5 | (–8%) | 0 | (1%) | –11 | (–6%) | –82 | (–22%) |
| Estonia | 8 | (12%) | 0 | (0%) | –0 | (0%) | –1 | (–3%) | –2 | (–11%) |
| Finland | — | | –1 | (–3%) | –0 | (–1%) | –1 | (–2%) | 5 | (21%) |
| France | –7 | (–1%) | –12 | (–2%) | 3 | (2%) | 15 | (1%) | –830 | (–21%) |
| Germany | — | | –5 | (–1%) | –5 | (–4%) | 13 | (1%) | –25 | (–1%) |
| Greece | — | | 1 | (1%) | 0 | (0%) | –1 | (–1%) | –28 | (–59%) |
| Hungary | — | | 4 | (6%) | –2 | (–5%) | 1 | (1%) | –327 | (–18%) |
| Ireland | — | | –0 | (0%) | 1 | (2%) | –1 | (0%) | –223 | (–41%) |
| Italy | –0 | (0%) | 6 | (2%) | –9 | (–10%) | –14 | (–2%) | –113 | (–1%) |
| Latvia | — | | –0 | (–1%) | –0 | (–1%) | –1 | (–2%) | 2 | (6%) |
| Lithuania | –8 | (–8%) | 0 | (1%) | 1 | (4%) | –2 | (–1%) | –146 | (–54%) |
| Luxembourg | — | | 0 | (1%) | 0 | (3%) | 0 | (0%) | –11 | (–7%) |
| Netherlands | — | | –8 | (–7%) | 0 | (0%) | 4 | (1%) | 38 | (4%) |
| Norway | 13 | (8%) | –1 | (–3%) | 0 | (1%) | –0 | (0%) | –164 | (–48%) |
| Poland | –7 | (–1%) | 4 | (1%) | 3 | (3%) | –12 | (–1%) | –579 | (–9%) |
| Portugal | — | | –0 | (0%) | 0 | (3%) | –1 | (–1%) | –91 | (–62%) |
| Republic of Macedonia | — | | 0 | (0%) | –0 | (0%) | –0 | (0%) | –1 | (–26%) |
| Republic of Moldova | –2 | (–4%) | 0 | (1%) | 0 | (4%) | –0 | (0%) | –125 | (–67%) |
| Romania | –35 | (–14%) | 4 | (2%) | –2 | (–2%) | –3 | (0%) | –338 | (–21%) |
| Russia | –189 | (–8%) | 43 | (6%) | 1 | (0%) | –18 | (–1%) | –1085 | (–43%) |
| Slovak Republic | –41 | (–31%) | 3 | (10%) | 0 | (2%) | –3 | (–3%) | 327 | (122%) |
| Slovenia | — | | 1 | (5%) | 0 | (3%) | 0 | (0%) | –61 | (–43%) |
| Spain | — | | –0 | (0%) | 2 | (2%) | — | | –541 | (–57%) |
| Sweden | — | | 0 | (0%) | 1 | (5%) | –4 | (–3%) | –124 | (–59%) |
| Switzerland | — | | –0 | (0%) | 0 | (0%) | 1 | (0%) | –6 | (–1%) |
| Ukraine | –159 | (–16%) | 25 | (5%) | 4 | (3%) | –26 | (–2%) | –1176 | (–40%) |
| United Kingdom | 56 | (5%) | –7 | (–3%) | 5 | (4%) | –14 | (–1%) | –1678 | (–22%) |
| Yugoslavia | –21 | (–23%) | 4 | (6%) | 0 | (1%) | –0 | (0%) | –79 | (–19%) |
| **Total** | **–426** | **(–3%)** | **54** | **(1%)** | **6** | **(0%)** | **–93** | **(–1%)** | **–8215** | **(–15%)** |

*Note:* Emissions are given as kilotons $NO_2$, $NH_3$, $N_2O$, and $CH_4$ /year; abatement costs are in million Euro/year; percentages represent a percentage of GAP$^{+}$ values.

[a] $SO_2$ emissions calculated for the GAP$^{+}$ and GAP$^{++}$ scenarios are the same for all countries.
[b] — indicates that there is no change; 0 indicates an increase smaller than 0.5; –0 indicates a reduction smaller than 0.5.
[c] Emissions from agricultural activities only.
[d] Total cost for technical measures to reduce emissions in Europe.

house gas emissions in the longer term. Several of these aspects can be included in the model, although for some of these extensions the linearised approach might be unsuitable. Major limitations are data availability (in particular data on side effects of abatement technologies) and also the size of the problem (because of the large number of abatement options that have to be included). The case presented in this study indicates that interrelations between different environmental problems affect cost-effective policies.

### ACKNOWLEDGEMENTS

This research was funded by the Netherlands Organisation for Scientific Research. We thank Dr. Jean-Paul Hettelingh for providing us with critical loads data for Europe. We thank two anonymous reviewers for their useful comments on an earlier version of the manuscript.

| | | |
|---|---|---|
| Received: | July | 13, 2001 |
| Revised: | September | 17, 2001 |
| Accepted: | October | 9, 2001 |
| Published: | October | 30, 2001 |

### BIOSKETCH

**Corjan Brink** is a researcher at the National Institute for Public Health and the Environment (RIVM), The Netherlands and is completing his Ph.D. thesis at the Environmental Economics and Natural Resources Group, Wageningen University, The Netherlands. His research deals mainly with cost-effectiveness of environmental policies and interrelations between policies for various environmental problems.
